# Respiratory Physiotherapy Interventions in Paediatric Population with Atelectasis: A Systematic Review

**DOI:** 10.3390/children11111364

**Published:** 2024-11-10

**Authors:** Carlota Beatriz Esteban-Gavilán, Patricia Rico-Mena, Javier Güeita-Rodríguez, Víctor Navarro-López, Raúl Escudero-Romero

**Affiliations:** 1 Department of Physiotherapy, School of Medicine, Universidad San Pablo-CEU, CEU Universities, Urbanización Montepríncipe, 28660 Boadilla del Monte, Spain; carlotabeatriz.estebangavilan@usp.ceu.es (C.B.E.-G.); raul.escuderoromero@ceu.es (R.E.-R.); 2 Neurosciences and Physical Therapy Research Group, Department of Physiotherapy, Faculty of Sports Sciences, Universidad Europea de Madrid, C. Tajo, S/N, 28670 Villaviciosa de Odón, Spain; 3 Research Group of Humanities and Qualitative Research in Health Science, Department of Physical Therapy, Occupational Therapy, Rehabilitation and Physical Medicine, Universidad Rey Juan Carlos, 28922 Alcorcón, Spain; javier.gueita@urjc.es; 4 Movement Analysis, Biomechanics, Ergonomics and Motor Control Laboratory, Department of Physical Therapy, Occupational Therapy, Rehabilitation and Physical Medicine, Faculty of Health Sciences, Universidad Rey Juan Carlos, 28922 Alcorcón, Spain; victor.navarro@urjc.es

**Keywords:** pulmonary atelectasis, paediatrics, physiotherapy, respiratory therapy

## Abstract

**Objective:** This systematic review aims to assess the effectiveness of respiratory physiotherapy techniques in oxygenation, chest X-ray findings, and lung auscultation in paediatric patients aged 0 to 18 years diagnosed with atelectasis. **Methods:** A comprehensive search was conducted in the PubMed, PEDro, Web of Science, and Cochrane Library databases. **Results:** Eight randomised clinical trials were included, involving 430 children ranging from 35 weeks of gestational age to 14 years. These trials evaluated various respiratory physiotherapy techniques and their effects on oxygenation and chest radiograph outcomes. The methodological quality of the studies ranged from acceptable to good, according to the PEDro scale. **Conclusions:** Recent evidence indicates that respiratory physiotherapy is effective and safe in the paediatric population with atelectasis. Both manual and instrumental techniques demonstrated efficacy, with instrumental techniques showing superior outcomes in many cases.

## 1. Introduction

Atelectasis is characterised by reduced pulmonary ventilation, often leading to localised or generalised lung tissue collapse. This collapse typically occurs in the lower airways, at the alveolar level, because of airway obstruction, decreasing the surface area available for gas exchange and compromising lung mechanics [1].

Children are more susceptible to atelectasis than adults due to the immaturity of their respiratory systems. Their underdeveloped airways are smaller and less functional, making them prone to collapse and respiratory complications [2]. Additionally, compensatory mechanisms, such as collateral ventilation through intraalveolar pores and bronchioalveolar channels [3], are less developed in early childhood, further predisposing them to atelectasis. Weak respiratory muscles and high compliance of the chest wall due to incomplete rib ossification also increase the vulnerability of paediatric patients to alveolar collapse [4].

Atelectasis is a pathological condition resulting from intrapulmonary and extrapulmonary disorders, representing a manifestation of underlying pulmonary diseases, including pneumonia, asthma, bronchiolitis, neuromuscular diseases, and cystic fibrosis [3].

The diagnosis of atelectasis is influenced by two key factors: the underlying disease and the extent of the airway obstruction [1,3]. Traditionally, common diagnostic methods include auscultation, chest radiography, and bronchoscopy [1,5]. While chest radiography remains widely considered the gold standard for detecting and evaluating atelectasis, recent advancements highlight the potential of lung ultrasound (LUS) as a valuable imaging tool, particularly for critically ill paediatric patients [6].

The primary goal in the management of atelectasis is to achieve a re-expansion of collapsed lung tissue, which is often pursued alongside treatment for the underlying disease [3]. In paediatric patients, there is no universally accepted gold standard for the management of atelectasis, and therapeutic interventions vary depending on the severity of atelectasis and the underlying pathology [1,3]. In the cases of acute pathologies with secondary atelectasis, complete resolution is usually often achieved within 2–3 months with conservative treatment management [1]. However, chronic atelectasis generally improves with a combination of pharmacological interventions and conservative respiratory physiotherapy [1,3,7]. When conservative approaches fail and symptoms worsen, surgical intervention may be required [1].

The goals of respiratory physiotherapy in this context include preventing respiratory complications, restoring or maintaining lung function, clearing airway secretions, expanding collapsed lungs, improving oxygenation, and enhancing overall quality of life [8,9]. Treatment of atelectasis in the paediatric population requires the use of specific techniques to address the lower clearance of airway secretion and lung re-expansion [10]. These techniques can be manual or instrumental and include postural drainage, slow inspiratory exercises (e.g., controlled inspiratory flow exercises), and various supportive devices such as volume incentive spirometers, positive expiratory pressure (PEP) devices, mechanical insufflation–exsufflation devices, and resisted inspiratory manoeuvres (RIMs) [8].

To date, there has been no systematic review assessing the effects of respiratory physiotherapy on paediatric patients with atelectasis. Therefore, this review aims to evaluate the efficacy of respiratory physiotherapy techniques in oxygenation, chest X-ray findings, and lung auscultation in children aged 0 to 18 years with atelectasis.

## 2. Materials and Methods

This review adheres to the guidelines outlined by the Preferred Reporting Items for Systematic Reviews and Meta-Analyses (PRISMA) [11]. The protocol for this review was not registered with PROSPERO (International Prospective Register of Systematic Reviews).

### 2.1. Search Strategy

Two independent researchers conducted a systematic search in May 2024 using the following databases: PubMed, PEDro (Physiotherapy Evidence Database), Web of Science, and the Cochrane Library. The search strategy incorporated Medical Subject Headings (MESH) and was adapted for each database (Table 1). Boolean operators “OR” and “AND” were used to combine the relevant descriptors.

### 2.2. Inclusion and Exclusion Criteria

Studies were included if they met the following criteria:

- Study type: Randomised controlled trials.
- Language: Studies published in Spanish, French, or English.
- Participants: Paediatric patients aged 0 to 18 years with atelectasis.
- Intervention: Respiratory physiotherapy techniques focused on the treatment of atelectasis compared to other interventions or standard care.
- Outcome measures: Oxygenation, chest X-ray findings, and lung auscultation.

Studies were excluded if the intervention was not performed by a physiotherapist, if the intervention occurred during surgery, if full-text access was unavailable, if the study focused on the prevention of atelectasis, or if the methodological quality was rated below 4 out of 10 on the PEDro scale.

### 2.3. Methodological Quality Assessment

The PEDro scale [12] was used to assess the methodological quality of the included studies, which assesses the criteria for randomisation, blinding, and data processing criteria. The total score ranges from 0 to 10, with studies rated as high quality if they score 6 or more, and low quality if they score 5 or lower [13]. Two independent authors assessed the methodological quality to ensure consistency, with disagreements resolved by consulting a third author if necessary.

The Cochrane Handbook for Systematic Reviews of Interventions [14] was used to assess the risk of bias, focusing on selection bias, performance bias, detection bias, attrition bias, reporting bias, and other potential sources of bias.

### 2.4. Extraction and Analysis

Data were systematically extracted using Microsoft Excel [15], capturing participant characteristics, interventions, and outcome measures. Two authors independently extracted the data, and any discrepancies were resolved through discussion or consultation with a third author if necessary.

Primary outcomes included chest X-ray findings and oxygenation levels, while secondary outcomes included lung auscultation. A narrative synthesis of the results was conducted due to the heterogeneity of the interventions and results, excluding a meta-analysis.

The outcome measures evaluated in this review included:

- Chest X-ray: Considered the gold standard for detecting and evaluating atelectasis. It was assessed either by using the Atelectasis Severity Index (ASI) or by an expert evaluation of the presence of radio-opaque areas in any lung field and the displacement of mediastinal structures.
- Oxygenation levels: Evaluated using measures such as pulse oxygen saturation (SpO_2_) or arterial oxygen saturation (SaO_2_), arterial partial pressure of oxygen (PaO_2_), or the Oxygen Saturation Index (OSI).
- Lung auscultation: Evaluated by experts, focusing on the presence or reduction in respiratory sounds in one or more pulmonary fields.

### 2.5. Synthesis and Analysis

In the qualitative analysis, studies comparing variables were included, considering measurements taken at baseline, after treatment, and during follow-up. The primary outcome of interest was pulmonary oxygenation, particularly the differences observed between pre- and post-intervention measurements. Secondary outcomes involved chest X-ray findings, such as the resolution of atelectasis.

Data on patient characteristics and interventions used, and the results were presented in tables, followed by a narrative synthesis. Although a meta-analysis was initially considered to aggregate data, it was deemed infeasible due to the heterogeneity in the outcome measures and interventions in the included studies. As a result, the findings were synthesised descriptively.

## 3. Results

### 3.1. Study Selection

**Figure 1:**
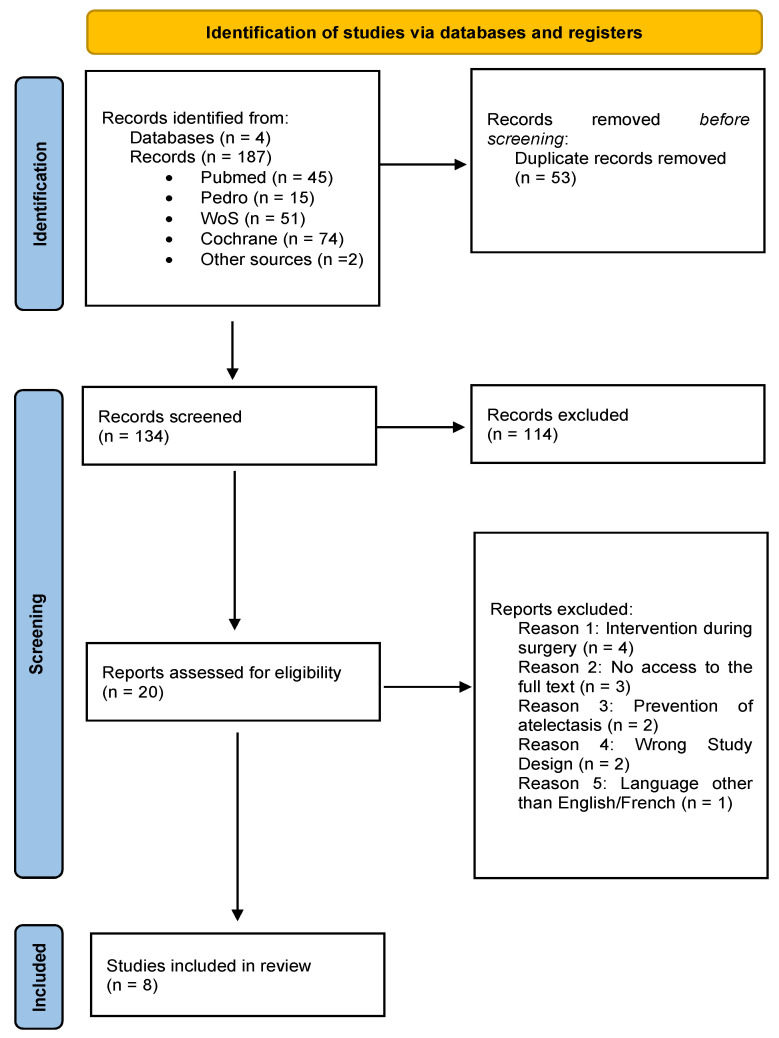
Flow chart adapted from the PRISMA 2020 statement: an updated guideline for reporting systematic reviews.

The search yielded 187 studies, 53 of which were duplicates. After screening titles and abstracts, 20 studies were considered potentially eligible. Full-text reviews led to the exclusion of 12 studies, resulting in the inclusion of eight studies in the qualitative analysis (Figure 1). The characteristics of the studies included in this systematic review are presented in Table 2.

### 3.2. Study Population

The included studies had a combined sample of 434 participants, with 231 in the intervention group and 203 in the control group. Although this review originally aimed to include studies with participants up to 18 years of age, the final selection included studies with patients from premature infants born before 35 weeks of gestation to 14 years old, as this was the upper age limit represented in the available literature. In four studies [16,17,18,19], birth weight was recorded, and in three, the type of delivery was also reported.

### 3.3. Study Variables

- **Chest X-ray**: This outcome was evaluated in three studies [19,20,21], which evaluated changes before and after treatment, revealing significant improvements in the resolution of atelectasis.
- **Oxygenation levels**: Six studies [17,19,20,21,22,23] analysed various oxygen saturation parameters, with significant differences observed between the groups, which favour the experimental groups. Furthermore, four studies [17,21,22,23] evaluated oxygen pressure, respiratory rate, and heart rate, with significant improvements observed between the pre- and post-treatment values, but not between experimental and control groups.
- **Lung auscultation**: This was used as a secondary outcome in only one of the included studies [21].

### 3.4. Intervention Methods

#### 3.4.1. Manual Techniques for Paediatric Atelectasis

Four studies [16,18,19,20] used manual physiotherapy techniques, including thoracic compression and percussion, to treat atelectasis.

The studies by Diniz et al. [23], Wong et al. [19], and Kole et al. [17] evaluated oxygen saturation before and after the interventions. Diniz et al. [23] reported a decrease in oxygen saturation, along with an increase in heart rate and respiratory rate in the experimental group after the intervention. However, Kole et al. [17] observed improvements in oxygen saturation, partial oxygen pressure, and arterial oxyhaemoglobin saturation. No significant changes in oxygen saturation were observed in Wong et al.’s study [19] after intervention.

Ashary et al. [20] evaluated the Oxygen Saturation Index (OSI), a non-invasive measure that continuously monitors oxygenation. Both the experimental and control groups exhibited decreases in OSI; however, chest X-ray findings indicated a greater improvement in the experimental group.

#### 3.4.2. Instrumental Techniques for Paediatric Atelectasis

Two studies [16,18] used instrumental techniques, specifically nasal continuous positive airway pressure (NCPAP), as a respiratory support intervention to manage atelectasis. NCPAP was associated with reductions in the duration of both oxygen therapy and NCPAP use.

Two additional studies [21,22] compared the effectiveness of instrumental techniques with manual respiratory physiotherapy. Siriwat et al. [21] compared the effects of the mechanical insufflation–exsufflation device (Cough Assist^®^) with manual respiratory physiotherapy techniques and found significant differences in the resolution of atelectasis, duration of treatment, and hospital stay. Similarly, Deakins et al. [22] compared intrapulmonary percussive ventilation (IPV) with manual physiotherapy techniques, noting significant differences in the resolution of atelectasis and oxygenation outcomes, which favours the use of IPV over manual techniques.

#### 3.4.3. Underlying Pathologies in the Paediatric Population with Atelectasis

Most of the studies included in this review diagnosed patients with respiratory distress or pneumonia along with atelectasis. The exceptions were the studies by Siriwat et al. [21], which included children with spastic cerebral palsy and acute lower respiratory tract infections, and by Pandita et al. [16], which included infants diagnosed with meconium aspiration syndrome.

### 3.5. Methodological Quality

The methodological quality of the included studies, assessed using the PEDro scale, ranged from a score of 4/10 to 8/10. The highest scoring study achieved an 8/10, while the lowest scored 4/10. Most studies scored between 5 and 7, reflecting a range from acceptable to good quality. The primary bias observed in all studies was the lack of blinding of participants and therapists, which is a common challenge in clinical trials involving physiotherapy interventions. The detailed scores for each study are presented in Table 3.

All studies exhibited some degree of bias, as determined by the Cochrane Handbook for Systematic Reviews of Interventions. The most frequent type of bias was related to the lack of blinding of therapists and participants. One study was identified as having a high risk of selection bias due to issues with both randomisation and allocation concealment [20]. Four studies demonstrated a high risk of bias in allocation concealment. In most other domains, the studies were rated as having either low or unclear risk of bias. Table 4 provides a comprehensive overview of the bias assessment for each study. 

## 4. Discussion

The available evidence on respiratory physiotherapy in paediatric patients with atelectasis remains limited. This systematic review includes eight studies with relatively homogeneous demographic characteristics, although interventions and outcome measures varied across the studies.

Regarding manual techniques, the study by Diniz et al. [23] concluded that thoracic compression techniques were not beneficial, as they worsened clinical symptoms and reduced oxygen saturation in infants . However, subsequent studies by Wong et al. [19] and Kole et al. [17] demonstrated that these techniques were both safe and effective, with significant improvements in oxygenation and re-expansion of collapsed airways observed after two weeks of treatment. Similarly, Ashary et al. [20] reported improvements in chest X-ray results after 10 days, despite no significant change in the Oxygen Saturation Index (OSI).

The duration of treatment may play a critical role in achieving therapeutic effects, as studies that implemented longer interventions, both in session length and in total number of sessions, showed better results compared to studies with shorter treatment durations. Additionally, evidence from other paediatric populations suggests that slow expiratory techniques may provide superior outcomes compared to conventional forced expiration techniques in children aged 0 to 24 months [24].

Regarding instrumental techniques, in the study by Kahramaner et al. [18], NCPAP was compared with nasal intermittent positive pressure ventilation (NIPPV) in premature infants diagnosed with respiratory distress syndrome. The results demonstrated that NIPPV significantly reduced both the duration of non-invasive ventilation and the incidence of atelectasis compared to NCPAP. Although NCPAP adjusted only for positive expiratory pressure (PEP) and oxygen fraction, NIPPV adjusted additional parameters such as positive inspiratory pressure (PIP) and the frequency of insufflations. Both modalities proved effective in preventing atelectasis post-extubation, but the incidence was statistically lower with NIPPV.

Furthermore, Komatsu et al. [25] reported that NIPPV was associated with a lower risk of extubation failure compared to NCPAP in preterm infants, although different ventilation parameters were used between studies. These findings suggest that NIPPV may offer greater advantages in preventing atelectasis and in facilitating extubation when the parameters are appropriately adjusted.

Deakins et al. [22] aimed to evaluate the effects of IPV compared to manual physiotherapy techniques. The study found that IPV significantly improved atelectasis scores compared to manual techniques, with shorter treatment durations and improved oxygenation outcomes. This suggests that IPV may be a more effective intervention for treating atelectasis in mechanically ventilated paediatric patients.

Both of the studies by Kahramaner et al. [18] and Deakins et al. [22], respectively, focused on intubated paediatric patients, where atelectasis is common due to factors such as weak respiratory muscles and pulmonary immaturity in premature infants or respiratory infections in older children. Evidence suggests that NIPPV is particularly effective in preventing atelectasis in preterm infants, while IPV may offer significant advantages in treating atelectasis in older children. More research is needed to explore the potential combination of these techniques for improving the prevention and management of atelectasis.

In paediatric patients with neurological impairments, the study by Siriwat et al. [21] compared a mechanical insufflation–exsufflation device (Cough Assist^®^) with a combination of manual techniques, including percussion, vibration, postural drainage, and assisted coughing. The mechanical insufflation–exsufflation device produced faster and more favourable outcomes, reducing hospital stays and treatment durations. These results align with existing evidence suggesting that the use of mechanical insufflation–exsufflation devices improves secretion clearance and reduces the risk of atelectasis or pneumonia in children with neuromuscular disorders [21].

Both the mechanical insufflation–exsufflation device and IPV demonstrated shorter recovery times compared to manual techniques, suggesting that instrumental approaches may be more effective in managing atelectasis in paediatric patients. Further studies are needed to evaluate the potential benefits of combining these instrumental techniques with manual physiotherapy.

Compared with other studies using NCPAP [18], the study by Siriwat et al. [21] used the EzPAP system for children with atelectasis, combined with a manual technique. While several positive expiratory pressure (PEP) devices exist, NCPAP with a PEP of 6 cm H_2_O continued to produce superior results in the management of atelectasis.

Despite differences in the underlying pathologies and the instrumental techniques employed, the studies by Pandita et al. [16] and Siriwat et al. [21] demonstrated significant improvements, reducing both hospital stays and the duration of oxygen therapy. The study by Pandita et al. [16] had a shorter duration, although the age of the patient, the underlying conditions, and the interventions differed between the two studies.

Except for the study by Diniz et al. [23], which concluded that respiratory physiotherapy had no beneficial effects on atelectasis due to worsening clinical signs and decreased oxygen saturation, all of the other studies included in this review suggest that respiratory physiotherapy, manual or instrumental, has positive effects in the prevention and treatment of atelectasis.

This systematic review has several limitations. First, the outcome measures used were not homogeneous across the studies, limiting the ability to compare the effects of different techniques. Second, the limited scientific evidence published to date required extending the range of publication years to conduct this review. There were also limitations in terms of diagnostic methods and progression of the condition, as only two studies [19,20] used chest X-ray as a primary variable to evaluate atelectasis. Consequently, more studies using this variable are required to objectively assess treatment outcomes. The absence of LUS as an outcome measure may also be a limitation, as it has been validated as a useful, radiation-free tool for monitoring lung conditions in critically ill children [6]. Therefore, future studies could incorporate LUS to objectively monitor treatment outcomes in paediatric patients undergoing respiratory physiotherapy for atelectasis. Lastly, all studies exhibited bias related to blinding, which should be acknowledged, although it is important to note that blinding is inherently difficult when applying physiotherapy techniques that require the active participation of the therapist.

## 5. Conclusions

The chest X-ray remains the most reliable diagnostic tool for detecting and monitoring the progression of atelectasis. Manual thoracic compression techniques may improve oxygenation when applied for 20 min in one to three sessions per day for 10 to 15 days. However, instrumental techniques, particularly NIPPV and IPV, appear to offer greater efficacy in reducing recovery times and improving outcomes in paediatric patients with atelectasis.

Further research is required to investigate the effects of respiratory physiotherapy on specific subgroups within the paediatric population, using standardised outcome measures to draw more precise and clinically relevant conclusions.

## Figures and Tables

**Table 1:** Search strategy for studies on respiratory physiotherapy in the paediatric population with atelectasis.

**Descriptors of Atelectasis**	**Descriptors of Population**	**Descriptors of Physiotherapy**
Pulmonary atelectasis Atelectasis	Paediatric Paediatric Paediatric	Physical therapy modalities Physiotherapy Respiratory therapy

**Table 2:** Description of the studies included in the review.

Author	Participants	Measures	Intervention	Results
Pandita et al. [16]	N = 135 (EG 67; CG 68) Newborns older than 35 weeks and weighing more than 2000 g admitted to the neonatal intensive care unit (NICU) for meconium aspiration syndrome.	Primary: Need for mechanical ventilation in the first 7 days of life Secondary: - Complications - Surfactant requirement - Duration of oxygen treatment - Hospital stay - Mortality	IG: nasal continuous positive pressure (NCPAP): - Starting NCPAP 5 cm H_2_O. - FiO_2_: adjusted to maintain oxygen saturation 90–95%. CG: standard care: - Oxygen in a hood administered at 5–10 L/min. Treatment was stopped when stable values were reached.	Need for mechanical ventilation: Lower in the IG during the first seven days (*p* = 0.002). Death, pneumothorax, sepsis, pulmonary hypertension: Higher in CG. Surfactant requirement: Higher in CG. Duration of oxygen treatment: Longer in CG. Hospital stay: Longer in the CG.
Kole et al. [17]	N = 60 (EG1 20; EG 2 20, CG 20) Premature babies aged 30–37 weeks admitted to the NICU with a diagnosis of respiratory distress syndrome or pneumonia.	Primary: - SpO_2_ - PaO_2_ - SaO_2_ Secondary: Chest X-rays: day 1 and last day	IG1: respiratory physiotherapy and reflex rocking. IG2: respiratory physiotherapy and chest compression technique. CG: respiratory physiotherapy. The three groups received three 20-minute sessions a day for two weeks. - Respiratory physiotherapy: manual thoracic percussion with the use of a neonatal face mask. - Chest compression technique: 3–4 chest compressions held for 5 s followed by a release phase. - Reflex rocking: light acupressure in the thoracic area (between the fifth and seventh ribs) while rotating the head to the side of the stimulus (four stimuli, two on each side).	SpO_2_ PaO_2_ and SaO_2_: There was an improvement in the three groups (*p* < 0.001), but there were no significant differences between the groups (*p* > 0.05).
Kahramaner et al. [18]	N = 67 (EG 39; CG 28) Premature infants <35 weeks and/or <2000 g birth weight diagnosed with respiratory distress syndrome.	- Post-extubation atelectasis - Extubation failure (re-intubation within 48 h of extubation) - Duration of intubation - Duration of non-invasive ventilation - Other complications	IG: nasal non-synchronised intermittent positive pressure ventilation (NIPPV): - PIP: 2 cm H_2_O. - PEP: 6 cm H_2_O. - Frequency: 25 breaths per minute. - FiO_2_: 0.4. CG: nasal continuous positive pressure (NCPAP): - PEP: 6 cm H_2_O. - FiO_2_.: 0.4. Treatment was discontinued when stable at minimal settings for 12 h.	Incidence of post-extubation and reintubation atelectasis: Lower in the IG than in the CG (*p* = 0.03 and *p* = 0.01). Duration of non-invasive ventilation: Longer in IG than in the CG (*p* < 0.001).
Wong et al. [19]	N = 56 (EG 26; CG 30) Newborns <37 weeks with collapsed lung.	- Chest X-ray: resolution of atelectasis - Recurrence of lung atelectasis - Duration of ventilation - Change in ventilator parameters - Hemodynamic parameters: heart rate, blood pressure (systolic, diastolic, and mean) and arterial oxygen percent saturation (SaO_2_) - Presence of pulmonary secretions - Other clinical outcomes: duration of ventilation, duration of oxygen dependency and the occurrence of bronchopulmonary dysplasia (BPD) - Intraventricular haemorrhage	IG: chest compression technique: - Three or four chest compressions held for 5 s followed by a release phase. - Application to both hemithoraxes in the supine position for 10 min followed by endotracheal suction. - Twice a day. CG: conventional percussion and vibration protocol: - Percussion and vibration manoeuvres in modified postural drainage position. - Application to both hemithoraxes for 10 min followed by endotracheal suction. - Twice a day.	Chest X-ray: Greater resolution of atelectasis in the IG. Recurrence of atelectasis: similar in both groups. Duration of ventilation: Shorter in the CG. Duration of oxygen dependency: Shorter in the IG. SpO_2_ : similar in both groups (*p* = 0.207).
Ashary et al. [20]	N = 44 (EG 22; CG 22) Children aged 6 months to 4 years with unilateral atelectasis and mechanical ventilation for more than 24 h.	- Oxygen Saturation Index before and after the intervention - Chest X-ray before the treatment and at the end of last session	IG: respiratory physiotherapy combined with chest compression technique: - Compression for 20 s on the healthy hemithorax during expiration, leaving the area of atelectasis free. - Invasive or non-invasive mechanical ventilation: PEP at 5–8 cm H_2_O for 20 min divided into 4 series of 5 min each. - 1 time/day for 10 days. CG: respiratory physiotherapy programme: - Modified postural drainage. - Percussion on the chest wall. - Mechanical vibration. - 1 time/day, 30 min for 10 days.	Oxygen Saturation Index: Decreased in both IG and CG (*p* > 0.05). Chest X-ray: Improvement in IG (*p* = 0.01).
Siriwat et al. [21]	N = 22 (EG 11; CG 11) Children with tetraparetic spastic cerebral palsy aged 7 months to 12 years with acute lower respiratory tract infection.	- Physiological parameters: body temperature, breathing frequency, heart rate, blood pressure, SpO_2_ and breath sound - Hospital lenght of stay. - Days of oxygen use - Therapy time - Chest X-ray - Prevalence of atelectasis	IG: mechanical insufflation–exsufflation: - A total of 3–5 insufflation/exsufflation cycles with an initial pressure of 15 cm H_2_O until a maximum pressure of 40 cm H_2_O insufflation/exsufflation of 2–3 s each. - Three times/day for 20–30 min. CG: manual respiratory physiotherapy: - A cycle of chest percussion, vibration, postural drainage, and manually assisted cough. - Subjects with atelectasis combined with nebulisation using EzPAP: 5 L/min initially until reaching the desired positive pressure + nebuliser 6–8 L/min of 100% oxygen. - One time/day. Duration of treatment: average 9–12 days.	Body temperature: Similar in both groups. HR: Similar in both groups. Respiratory rate: Higher in IG (*p* = 0.40). Blood pressure: Similar in both groups. SpO_2_: Similar in both groups. Breath sound: Similar in both groups. Hospital lenght of stay: Longer in the CG (*p* = 0.12). Days of oxygen use: Similar in both groups. Therapy time: Shorter in IG (*p* = 0.01). Chest X-ray: Improved in both groups after treatment. Prevalence of atelectasis: Shorter treatment time in the IG (*p* = 0.01).
Deakins et al. [22]	N = 12 (EG 7; CG 5) Children between 7 weeks and 14 years of age with atelectasis intubated and mechanically ventilated.	- Atelectasis score - Static compliance - SpO_2_ - Respiratory rate - Treatment duration to the resolution of atelectasis (days)	IG: intrapulmonary percussive ventilation: - Maximum observed pressures 15–30 cm H_2_O. - Frequency: 180–220 cycles/min. - 10 min every 4 h. CG: respiratory physiotherapy: - Percussion, clapping, and vibration manoeuvres in the areas of atelectasis. Suction at the end of the session. 10–15 min every 4 h.	Improvement in atelectasis score: Greater in IG (*p* = 0.026). Static compliance: Similar in both groups. SpO_2_: Similar in both groups. Respiratory rate: Similar in both groups. Treatment Duration to the resolution of atelectasis: Lower in IG (*p* = 0.018).
Diniz et al. [23]	N = 38 (EG 19; CG 19) Children between 29 days and 24 months with a diagnosis associated with atelectasis (IG) versus absence of respiratory disease (CG).	- Signs of respiratory distress - Respiratory rate (timed for 1 min) - HR - SpO_2_	IG: chest compression technique: - Hand on the healthy hemithorax with symmetric pressure in an oblique direction from top to bottom. - Application during the expiration phase for 10 s with a total of 6 repetitions of the manoeuvre for 60 s. - Duration of the technique: 5 min. CG: chest compression technique: - Hand on the healthy hemithorax with symmetric pressure in an oblique direction from top to bottom. Application during the expiration phase for 10 s with a total of 6 repetitions of the manoeuvre for 60 s.	Signs of respiratory distress: Higher in IG (*p* > 0.05). HR: Higher in IG *. Respiratory rate: Higher in the IG. SpO_2_: Lower in IG *. * There is a positive correlation between SaO_2_ and HR in IG after the techique.

IG: intervention group; CG: control group; *p* < 0.05 statistically significant; SpO_2_: pulse oxygen saturation; SaO_2_: arterial oxyhaemoglobin saturation; PaO_2_: partial pressure of oxygen; HR: heart rate; PEP: positive expiratory pressure; FiO_2_: fraction of inspired oxygen; PIP: positive inspiratory pressure.

**Table 3:** Scoring of the included studies with the PEDro scale.

Study	Item 1	Item 2	Item 3	Item 4	Item 5	Item 6	Item 7	Item 8	Item 9	Item 10	Item 11	Total
Pandita et al. [16]	1	1	1	1	0	0	0	1	1	1	1	7/10
Kole et al. [17]	0	1	1	0	0	0	0	0	0	1	1	4/10
Kahramaner et al. [18]	1	1	1	0	0	0	0	1	0	1	1	5/10
Wong et al. [19]	1	1	1	1	0	0	1	1	1	1	1	8/10
Ashary et al. [20]	1	1	0	1	0	0	0	1	0	1	1	5/10
Siriwat et al. [21]	1	1	0	0	0	0	0	1	0	1	1	4/10
Deakins et al. [22]	1	1	1	1	1	0	1	1	0	1	0	7/10
Diniz et al. [23]	1	1	0	1	0	0	1	1	1	1	1	7/10

**Table 4:** Risk of bias of the studies included in the scale proposed by the Cochrane Handbook. 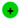 Low risk of bias. 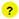 Unclear risk of bias. 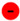 High risk of bias.

	Random Sequence Generation (Selection Bias)	Allocation Concealment (Selection Bias)	Blinding of Participants and Personnel (Performance Bias)	Blinding of Outcome Assessment (Detection Bias)	Incomplete Outcome Data (Attrition Bias): All Outcomes	Selective Reporting (Reporting Bias)	Other Bias
Pandita et al. [16]	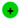	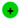	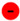	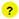	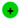	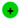	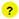
Kole et al. [17]	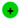	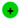	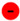	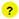	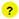	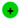	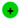
Kahramaner et al. [18]	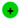	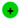	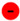	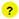	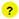	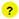	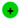
Wong et al. [19]	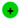	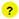	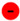	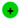	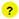	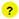	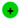
Ashary et al. [20]	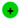	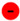	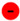	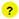	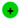	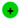	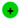
Siriwat et al. [21]	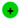	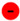	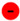	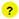	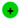	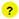	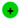
Deakins et al. [22]	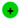	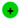	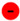	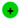	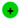	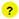	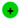
Diniz et al. [23]	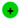	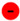	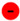	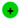	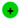	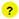	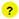

## Data Availability

The datasets generated and/or analysed during the current study are not publicly available due to ethics restrictions but are available from the corresponding author on reasonable request.
